# Processes and Experiences of Creative Cognition in Seven Western Classical Composers

**DOI:** 10.1177/1029864920943931

**Published:** 2020-08-08

**Authors:** Andrea Schiavio, Nikki Moran, Dylan van der Schyff, Michele Biasutti, Richard Parncutt

**Affiliations:** University of Graz, Austria; The University of Edinburgh, UK; The University of Melbourne, Australia; University of Padova, Italy; University of Graz, Austria

**Keywords:** Musical creativity, composition, exploration, body, interaction

## Abstract

In a qualitative study, we explored the range of reflections and experiences involved in the composition of score-based music by administering a 15-item, open-ended, questionnaire to seven professional composers from Europe and North America. Adopting a grounded theory approach, we organized six different codes emerging from our data into two higher-order categories (*the* *act of composing* and *establishing relationships*). Our content analysis, inspired by the theoretical resources of 4E cognitive science, points to three overlapping characteristics of creative cognition in music composition: it is largely exploratory, it is grounded in bodily experience, and it emerges from the recursive dialogue of agents and their environment. More generally, such preliminary findings suggest that musical creativity may be advantageously understood as a process of constant adaptation – one in which composers enact their musical styles and identities by exploring novel interactivities hidden in their contingent and historical milieux.

The present contribution offers an account of creative cognition in music composition that is grounded on exploration, bodily experience, and interaction. We base such insights on the results of an original qualitative study with Western music composers, and on the conceptual tools of 4E cognitive science, a school of thought that sees mental life as an Embodied, Embedded, Extended, and Enactive phenomenon (Newen et al., 2018). Scholars working within 4E cognitive science argue that cognition is not reducible to in-the-head processes, but rather involves the whole living body, as well as its social and cultural niche (e.g., Di Paolo et al., 2017; Fuchs, 2018). This aligns well with recent scholarship that places emphasis on the collaborative roots of musical creativity (see Bishop, 2018) and at the same time challenges the focus on the composers’ individual activity and innate talent central to “the mythologised discourses of classical music” (Cook, 2018, p. 73). The romantic *myth of the lone genius* (Montuori & Purser, 1995) has strongly influenced popular conceptions of classical composers. Conceived of as heroic, gifted, and typically white, male individuals, classical composers are thought to use their exceptional intellectual abilities to create music in isolation before committing its authoritative form to paper. This depiction of musical creativity is partial at best. It is certainly not typical, to say nothing of the inequalities that such stereotypes perpetuate. In reality, the range of experiences involved in composing music also includes lower-level, visceral processes, as well as social contingencies and extra-musical concepts. These can affect the creative trajectory in various ways, and may lead to unexpected musical outcomes. To provide concrete examples of how this complex web of factors takes part in creative cognition, we designed a qualitative study with expert composers. They were asked to provide verbalizations of their experiences, and descriptions of how emotional, ecological, and bodily-based contingencies contribute in shaping their practice. In what follows, we first offer an overview of 4E cognitive science and its main tenets; we then map these insights to creativity research focused on music and music-making. In the subsequent section we present our qualitative study, and discuss its principal results through the lenses of this cross-disciplinary account.

## What is 4E Cognition?

The mind-as-computer metaphor that pervaded early work in cognitive science has arguably lost most of its charm due to its limited explanatory role in understanding lived experience and mental life more generally (Thompson, 2007; Varela et al., 1991). Indeed, many researchers have become increasingly fascinated by the view that our ability to think, act, and generate meaning is rooted in the complex coupling between brain, body, and world and, as such, not reducible to head-bound processes (Chemero, 2009; Gallagher, 2017). This shift in focus promoted the emergence of research situated under the umbrella term *4E cognition*: the view that mind is Embodied, Embedded, Extended, and Enactive. It is *embodied* because our body plays a constitutive role in shaping thought and action (Shapiro, 2011). For example, we often ground categorizations of abstract terms into metaphors associated with bodily experience (Lakoff & Johnson, 1980), and we use our body to facilitate processes such as reasoning, calculation, and memory (Clark, 2008). This work inspired research investigating the action-dependent roots of musicality (Leman, 2007; Leman & Maes, 2016; Schiavio & Altenmüller, 2015), examining, among others, how musical learning is intrinsically related to action (e.g., Bowman, 2004), how more accurate musical memories can emerge through active engagement with the musical material when compared to passive listening or observation (e.g., Schiavio & Timmers, 2016), and how music perception is modulated by the listener’s degree of motor expertise with a musical instrument (e.g., Overy & Molnar-Szakacs, 2009). The qualitative study reported in the next section picks up a related thread within the discourse of 4E music cognition research to examine how our body shapes the creative reach of the composer in a number of ways.

Through our bodies, indeed, we explore, shape, and transform the ecological niche in which we are situated, affecting in the process how we think and feel. Cognition, in other words, is also *embedded*: the patterns of sensorimotor activity we adopt to engage with our environment derive from, and help develop, our cultural and social presence (see Malafouris, 2013; 2015). On both evolutionary and ontogenetic time-scales, many contributions point to the co-dependencies of living systems and their niches (e.g., Oyama, 2000), leading to a novel understanding of the interplay between subjects and their world. This view has important similarities with research that emphasizes the cultural roots of musical experience, as well as the social aspects associated with musical production and development (Krueger, 2013; Morrison et al., 2003; 2008; Schiavio et al., 2018). If music is inherently social (see Mithen, 2006; Small, 1998; Turino, 2008), then it involves a kinaesthetic, dialectic, shared experience with others; as Cross puts it:

Music’s embodied characteristics may provide the basis for music’s capacities to coordinate and entrain action in time . . . Music is embedded in social action, deriving meaning from that action and in turn endowing it with significance. (Cross, 2003, p. 108, quoted in Moran, 2014)

The social aspect of music, as we will see in our qualitative study, is well understood as a resource for developing inspiration and creativity. However, speculation based on a 4E story suggests that it may also involve a deeper layer of causation: creative outcomes, it might be assumed, are co-determined by a social, imaginative, ecological dialogue; and if so, we would expect composers to enact this in their practice.

Among the many patterns of interactivity that music and music making help establish within a broader sociocultural niche, those developed socially, with others, are the most apparent. Yet these do not account for all possible types of relationships. Indeed, musicians often form hybrid relationships with the instruments they play and the technologies that surround them^1^ (see Schiavio et al., 2020); as such, those instruments and tools can become an extension of the musician’s body (Nijs, 2017). The integration of biological and non-biological systems described here is often explored through the theory of *extended* mind (Clark & Chalmers, 1998). In its original form, this approach is best captured by the parity principle: “if, as we confront some task, a part of the world functions as a process which, *were it done in the head*, we would have no hesitation in recognizing as part of the cognitive process, then that part of the world is (so we claim) part of the cognitive process” (Clark & Chalmers, 1998, p. 8, emphasis in original). As we will see, compositional practices often involve a fluid integration of internal and external resources, including those provided by tools such as pencil and paper, or computers, as well as musical instruments and their associated techniques.

The *enactive* dimension brings together the main ideas presented here – the key role of body and socio-material environment for mental life – and explores the dialogue between the autonomy of individual organisms (their ability to sustain themselves under precarious conditions), and their situated activity (Varela et al., 1991). Living systems, on this view, develop their identity through a *history of structural coupling* with the world. This feature fosters sensorimotor adaptations adequate for the system to engage with the environment’s perturbations in a meaningful (i.e., conducive to survival and well-being) way. These adaptations can take multiple forms, and involve different values, activities, and normative domains. In a musical context, for example, one can explore how the meanings one develops in performance are transformed by the community of practice in which musicking takes place (see Kenny, 2016; Small, 1998; Wenger, 2002). This gives rise to a constant negotiation of individual and collective agencies, experiences and identities, which shape the sonic ecologies being instantiated in the act of musicking (Loaiza, 2016; Reybrouck, 2005; 2012).

In what follows, we first connect these insights to recent creativity research; we then present our empirical study to offer concrete examples which can help situate these considerations into a concrete context.

### Musical Creativity: Exploration, Bodily Experience, and Interaction

Creative products – ideas, items, musical compositions – usually display an optimal balance between *originality* and *appropriateness* (Runco & Jaeger, 2012), involve a good deal of *surprise* (Boden, 2004), and are fully *realized* in a given setting (Mackinnon, 1965). As Bishop (2018), suggests, achieving good equilibrium between originality and appropriateness in musical contexts might involve the ability to “maintain flexibility within a given set of stylistic constraints”. A composer can thus be seen as creative if their compositions maintain recognizable features associable with a particular genre – say, Brazilian *choro* – while implementing novel structural, instrumental or harmonic configurations. The element of surprise can thus emerge from the combinatorial nature of the genre of choro itself, which brings together elements of European classic dances (such as polka, or gavotte) with rhythmical and expressive forms associated with traditional South American music (such as *tango brasileiro*). This musical elision can be realized through a rich variety of ensemble settings (with differences in terms of size and instrumentation), or through the re-creation of particular forms. Examples can be found in two famous works by Brazilian composer Heitor Villa-Lobos (1887–1959), *Suite Populaire Brésilienne* and *Bachiana Brasileira*, which allowed the composer to integrate Western compositional techniques (e.g., counterpoint) and forms (e.g., fugue), with Brazilian folk styles and traditions.

More than three decades ago, Sloboda (1985) noted that contemporaneous research dedicated to creativity in composition has sought to account for its products rather than its processes. Insights from musical analysis, for example, may provide precise characterizations of the underlying mechanisms governing a composition’s internal coherence and form, revealing what critical role different structural elements might play for the economy of a given piece of music. This, in turn, can help researchers to clarify the extent to which a musical piece integrates existing styles, and what original factors can be found in it (as we just saw in the case of *choro*). While productive in many ways, one consequence of this somewhat closed loop of musicological discourse is simply to demonstrate an endorsement – or censure – of the creative credentials of a particular composition. Since Sloboda’s observations 35 years ago, swathes of process-focused (ethno)musicological research have led to some deliberate moves to diversify musical analyses, such as the establishment of the *Journal of Analytical Approaches to World Music* in 2011. These have contributed to a broader perspective on creativity in musical composition.

Yet there remains a dearth of literature on the cognitive activity that composing musicians enact in the process of creating new music. As pointed out by Collins (2005), the norm for studies concerned with composers is to adopt an autobiographical approach, where reported perspectives and experiences are examined through various different theoretical or computational frameworks (see for example Webster, 1992). This reflects the major difference between research within scientifically-driven domains such as music perception, where the objects of study (e.g., stimuli, physiological reactions, etc.) can be quite easily conceptualized and isolated, compared to scholarship in the domain of musical composition, where the units of analysis remain seemingly ineffable. What this can foster is a close focus on the rich variety of processes involved in creative cognition, allowing scholars to generate novel taxonomies of factors, experiences, and concepts which are proposed to help explain the generation of musical ideas. Generally speaking, a classic example can be found in the work by Wallas (1926), who posited a four-stage model based on (i) the acquisition of knowledge, (ii) the elaboration of this knowledge, (iii) the sudden realization of a creative outcome and (iv) the evaluation in context of the specific item being generated. A similar approach was put forward by scholars supporting the *Geneplore* (“generate and explore”) model (Finke et al., 1992). As Abraham (2018) explains, this involves a first, generative phase, where original ideas are developed in a context-dependent manner: they can be “simple or complex, conceptually focused, or relatively ambiguous” (p. 65). This phase is followed by an exploratory step, where ideas and concepts are “evaluated in terms of their usefulness and feasibility” (ibid.). As both phases are associated with processes of discovery, creators may go back and forth between generative and explorative procedures, and alternate thinking with verification until a satisfactory outcome is found.

The notion of *exploration* is particularly suited to help capture the creative aspects involved in music-making and compositional practice. This aligns with recent work by Høffding & Schiavio (2019). Here, it is argued that music-making is best understood as an exploratory activity engaging with intimate states of mind (such as private emotions, memories, motor skills, imagination), as well as the (physical, social and cultural) environment in which musicking unfolds (see also Malloch & Trevarthen, 2018). From a creativity-focused perspective, the idea echoes previous work by Brown and Dillon (2012), who suggest that

… creative acts can be considered in two complementary dimensions: (1) types of action and the extent of engagement with music through them, and (2) contexts for action and the opportunities for meaning they provide. Through these lenses, the experience of meaningful engagement involves an immersion in a creative process that provides the composer to connect with his or her intuitive experience, or ‘acquainted knowledge’ of music. (p. 79)

Recent empirical work on musical collaboration increasingly explores the layers of reciprocal interaction between performers and the musical contexts they develop and transform (see e.g., Bishop, 2018; Demos et al., 2018; Walton et al., 2018). Yet the interplay of bodily, experiential, and environmental aspects associated with creative development remains only partially examined in current literature: traditional research in the broader field of creativity studies is often concerned with smaller units of analysis – for example, computational and neuronal processes. Here, differences at the level of information-processing are explored as associative combinatorial activity and subsequent development of mental representations (see e.g., Mednick, 1962). By this view, a creative person is someone who can easily integrate different concepts: their semantic network is characterized by a strong flexibility, which enables conceptual manipulations and access to diverse representations (see Kenett et al., 2014). To account for the impressive variability of creative thought and action, this general orientation posits a number of intra-individual models: Abraham (2018), for example, describes the following cognitive operations as fundamental aspects of creative processing: insight; analogy; metaphor; imaginary; conceptual expansion; the overcoming of knowledge constraints; and flow (pp. 67–74). These categories are largely influenced by the cognizer’s body and their surrounding environment: for example, one can observe how the use of gross and fine motor skills are always present in musicking, grounding the composer’s intentions and musical outcomes into bodily experience, perhaps via kinaesthetic memory^2^ (Burnard, 1999, pp. 170–171; reported in Abraham, 2018, p.187). Such an idea brings to mind the enactive notion of *structural coupling*, briefly presented above: organisms develop their concerned perspective via motivated patterns of action situated within a contingent milieu. Whilst such a repertoire of action is sedimented in one’s kinaesthetic memory, it must be flexible enough to be re-explored and re-constituted, leading to a functional stability that is conducive to well-being; similarly, creative thought in composition might be distributed between resources associated to the body in action (e.g., the instrumental expertise one might have, and which could be further explored) and relevant aspects of the environment one engages with.

This brings us to the notion of *interaction*, which is increasingly being addressed by scholars interested in collaborative forms of creativity (Sawyer & DeZutter, 2009). Seddon and Biasutti (2009), for example, examined distinct forms of interactivity in musical ensembles based on instruction, cooperation, and collaboration. The first one involves the transmission of knowledge between members of the group, the second is concerned with open communication and exchange of knowledge among musicians, and the third describes the interdependent chains of actions that lead the ensemble to explore novel musical possibilities (see also Biasutti, 2015; 2018). As such, group creativity might be understood as a “goal-oriented social interaction” (Hosseini et al., 2019), where meanings and action-possibilities are negotiated among peers as the task unfolds. This stands in contrast with the more traditional approaches to creativity described above, which reflect “larger trends in Western culture of leaning toward individual agency” (Hill, 2018, p. 73). The latter, as Cook has argued, “is a defining tradition of western ‘art’ musical culture, the very tradition that Sawyer called ‘the one remaining bastion of the solitary lone genius myth’” (Cook, 2018, p. 65). Indeed, while we can offer many examples of studies specifically focused on collaborative creativity in performance and music improvisation (e.g., Bailey, 1993; Burrows, 2004; Canonne & Garnier, 2015; Linson & Clarke, 2017; Sawyer, 2006; Toop, 2016; Walton et al., 2014), less is known about the kinds of collaborations and interactions involved in musical composition. The “cultural significance often attributed to individual [composers]” (Bennett, 2012, p. 146) may well have discouraged, until recently, the application of this socially curious approach to expert compositional practice. Yet a series of fascinating studies with undergraduates and young children involved in collaborative compositions (e.g., Barrett, 1998; Kratus, 1989; MacDonald et al., 2000; Morgan, 1999), and recent work in peer-to-peer music education, emphasise the role of reciprocal interaction for musical development and flourishing (see Johansen & Nielsen, 2019). Other work in the field of skill acquisition points to a distinctive role of sociality even in seemingly individual situations (Schiavio, Gesbert et al., 2019; see also Høffding & Satne, 2019). Here, the idea is that skills are always negotiated within a community of practice, and therefore cannot be nurtured or enhanced without its involvement. Similarly, composers do not operate in a vacuum; their musical ideas and choices emerge from a constant dialogue with their cultural and social environment. This interaction, however, is difficult to capture, playing out at different layers of awareness: some composers are perfectly conscious of the influences and inspirations they obtain from different ecological factors, while others might be less prone to recognize or articulate their experience.

In what follows, we report a qualitative study that explicitly addresses the challenges described here. In doing so, we (partially) heed the call of Sloboda (1988), who suggested that “the only way to understand cognition in composition (particularly professional composition) is to discuss out loud during direct engagement with such wide-ranging tasks” (quoted in Dean, 2017, p. 258). This *real-time* approach has been proven useful to capture important aspects of creative experience (see Collins, 2005), and can help us ground in concrete examples the preliminary insights developed here. What do composers experience in their practice? How can they relate to things and people within their environment? And what kind of connections can they establish between their ideas, concepts, memories and emotions?

## Methods

### Participants

Participation in the study was voluntary. It was supervised by the research team after ethical approval was granted by the ethics committee of the University of Graz, Austria. In total, 15 composers were recruited initially after an announcement was posted on social media. The sample was then reduced to seven individuals (one female; six males; median age 45.28 years (range: 29–73 years) who met the following inclusion criteria: having a higher degree in music composition, having composed at least 40 pieces; and being an active composer. Participants had studied at music schools and universities in Europe and North America; they were all highly familiar with Western classical and contemporary repertoires; all were expert performers. The main instruments they played were piano (*n* = 5), bass clarinet (*n* = 1) and horn (*n* = 1). After agreeing to participate in the study, they received two documents via email: a consent form to be signed and returned to the researchers, explaining the procedure and use of their data, and the questionnaire described below.

### Questionnaire

An open-ended questionnaire developed by AS, MB, and DvdS was sent via email to all participants, in English or Italian according to the participant’s native language. It comprised an initial section that focused on participants’ demographics and musical backgrounds, and a second section, dedicated to their practices and experiences as composers (see Appendix). This consisted of 15 open-ended questions to which participants were instructed to respond freely, discursively and without word limits. The written responses included examples to demonstrate participants’ compositional styles and techniques, evaluations of their creative practice, and personal reflections on their musical identity. The questionnaire items were deliberately generic and unspecific, so as to elicit a wide variety of answers and descriptions. A similar approach had been used successfully in previous studies investigating the teaching and learning of music in collective and individual settings (Schiavio et al., 2018; Schiavio, van der Schyff et al., 2019).

### Data Analysis

**Figure 1.**
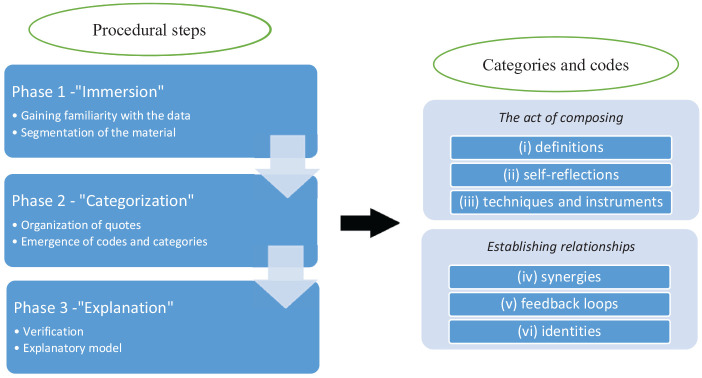
Scheme of the data analysis, including codes and categories.

Data were analysed using a grounded theory approach. This is an inductive method based on content analysis, in which relevant categories are derived directly from the dataset. This approach has been implemented in previous music research focused on education, pedagogy, and performance among others (e.g., Biasutti, 2013). To assist the analytical process, qualitative research analysis software, ATLAS.ti 7, (Scientific Software Development GmbH) was used to support data coding. The analysis involved three main steps: *immersion, categorization*, and *explanation*. The process started with an immersion in the raw data set, where initial reflections were discussed by the researchers as familiarity with the breadth of responses was gained. In this phase, relevant quotations were extracted, segmented, and translated into English when necessary. In the second phase, the selected quotations were organized systematically around concepts, and codes were assigned to groups of answers. The process was repeated several times to avoid redundancy, giving rise to a total of six codes: (i) definitions; (ii) self-reflection; (iii) techniques and instruments; (iv) synergies; (v) feedback loops; and (vi) identities. Two higher order categories were established: *the act of composing*, consisting of codes (i), (ii) and (iii), and *establishing relationships*, consisting of codes (iv), (v) and (vi). This classification was discussed by the research team; different interpretations were compared until a final agreement was reached and a preliminary explanatory model developed. Figure 1 summarizes the analytical process, and presents the codes that were identified and the categories into which they were clustered.

## Results and Discussion

### The Act of Composing

This section focuses on a number of important aspects and descriptions associated with the compositional practices and styles of our participants. These include statements regarding their definitions of *composing music*, reflections on their own feelings and emotional states while composing, as well as information about their intimate connections with musical instruments and their techniques.

#### Definitions

Musical composition within what might be described as the broad church of Western classical music involves the use of different forms, cultures, styles, and methods. One initial insight representing the first code is captured by the tension between the notions of *process* and *result*: “Composing music is the act of writing music in a way that employs compositional techniques and processes in order to devise and develop ideas. For me, a composition is often the end result of a learning process” (P. 01); “I think the ‘sense’ of my practice dwells in the very act of composing, without thinking of it as an outcome or as a result that transcends the act which starts its constitutive processes” (P. 02). The first quotation suggests that composing music might be understood as the final outcome of a creative activity. Conversely, the second quotation highlights the crucial role of the process itself. These two views, however, do not necessarily stand in opposition to each other. The desire to *produce* a particular item, appears associated with the sensation of absorption in the creative *process*. The following excerpt, for example, points to the recursive interplay between personal growth and the development of a particular mental state:

[Composing music] means expressing my desire to make things, and to be in a mental state of making, which I really enjoy. This mental state feels essential to the personhood I’ve constructed. It is important to me to always be “in the middle” of a project, and the process of composing is a way of being in the middle. (P. 03)

Such a state of mind, as reported, can even help the composer understand, re-live and re-conceptualize previous experiences and emotional states: “Composing is also a means to explore feelings and knowledges that I encounter in my life, and to create new ways of conceptualizing life. It’s a sort of playground for testing and creating ideas” (P. 03). The act of composing music can bring together past experiences and moment-to-moment, present, aesthetic choices and feelings, in a unique, structured, dialogue. This organic set of past and present dimensions may lead to broader conceptions of what composing music entails. In a sense, composing music can allow the composer to (re-)establish connections with things, places, ideas, and people. This was explained in more detail by another participant:

For sure composing is a practice that is learned at some stage, and this may take so much time and energy that in the end it might be the only thing that a composer is able to do as a job. So, composing is also a job, possibly. But composing is also a way to connect to music, that is, to an element that is way more abstract and impalpable than money, reputation and other social constructs, but at the same time is directly rooted on mechanical properties of matter so that it might give the impression of being docilely founded on physical science. And nevertheless, it has the ability to “teleport” us, even if for a moment, to a distant or deeper universe, in which . . . our daily reality instantly disappears. To compose is to learn, experiment and explore this teleporting device. (P. 04)

This understanding of music as a teleporting device aligns with research that conceives of musical activity (e.g., music listening) as “something that defies the mundane” (Frith, 1996, p. 275) or that inspires forms of transcendence (see Fachner, 2008; Schäfer et al., 2013). Yet the quotation reported here emphasizes more quotidian, relational connotations, in the associations with experiences, people, and sounds, that emerge from the act of composing. Interestingly, music is not seen here as a simple outcome that emerges from a specific process; rather, it is seen as a functional element of this relational interplay; the composer *engages with* music, or *connects to* music, rather than generating it. In what follows we focus on the range of bodily experiences and emotions associated with this dynamic process.

#### Self-Reflections

In this code, we include descriptions of bodily feelings, states, and sensations while composing, reported by our participants. The following three quotations, from different participants, suggest that composing is not always a joyful process; if things are not going as planned it can involve unpleasant sensations associated with uneasiness and stress:

When it’s going well, it’s thrilling. A little like being intoxicated; there’s a “rush” that I feel when it’s all clicking the way it should. When it’s not, it’s depressing. There’s a feeling of helplessness and failure. I’ve learned to shake off those negative feelings and trust that the right solutions will present themselves if I’m patient. (P. 05)

When things are going well, I feel good. When things are going badly (as they do, more often than not) I tend to feel pretty lousy, exhausted, stressed, and anxious. I rarely find composing to be a fun activity, yet I feel compelled to do it. (P. 01)

I feel excited when I build up the technique, the constellation of ideas on which I organize my work (a process that can take days or even months). Uncomfortable and impatient when I’m in the process of realizing it as a score, so that I never want to interrupt writing until I finished it. And kind of anxious to hear the result whenever the score is complete. (P. 04)

Composing music can always involve a wide variety of feelings and emotions, ranging from excitement to exhaustion. There is also a clear causality between the success of the activity and the feelings it evokes. As one participant put it: “I am focused on the piece in development and what I feel depends on how the creative process unfolds” (P. 02). This last quotation stresses again the uncertainty inherent in the process, and connects it with attentional focus and its associated range of feelings. The next excerpt, however, develops a more general statement:

I suppose my dominant compositional feeling is a kind of quiet contemplation; I feel lost in the process, like time is standing still; I won’t notice that the sun has set and it’s completely dark in the room where I’m playing (this happens to me all the time). Composing calms me down and takes me to a peaceful place, that’s also occasionally fun or more deeply rewarding. (P. 03)

The idea of being “taken somewhere” by the music or – in the words of another participant – being “teleported” elsewhere seems to be an important factor. Music provides access to an intimate sphere of feelings that are not easily mapped onto the state of things occurring in the composer’s surroundings.^3^ Importantly, this does not imply a lack of connections to the present environment. Indeed, at least two important *relational* aspects can compensate for this absence of direct mapping:

It often feels like I cohabit with an external entity that claims its independency, following its own . . . itinerary of individuation, growth and determination. Then I suddenly realize I am deeply pervaded with the music under development. I am . . . convinced that musical creation constitutes a primary way to access a pre-personal, or trans-personal, level of experience. (P. 02)

Sometimes I feel a desire to compose but don’t have any ideas, or am unmotivated. Most of the time, though, I compose automatically, just because the piano is nearby and I can’t resist touching it, or I hear something I like and I want to play with it and modify it. (P. 03)

The first quotation echoes the previous comments on the bi-directional relationship between composers and their music. A process of reciprocal causation arguably permeates the compositional moment, where both music and composer shape each other. Moreover, not only do composers engage with music itself and its properties; instead, they also develop intimate forms of reciprocal interplay with musical instruments, as the second quotation indicates. In what follows we aim to explore these processes of co-transformation and interaction in more detail.

#### Techniques and Instruments

This code includes experiences and descriptions of the various forms of engagement our participants develop with both the musical instruments they compose for and those that support their creative process. It also provides information concerning the compositional techniques employed, and how these can inform their artistic activity. Let us first consider the tools used by our participants to aid their compositional processes. The following two quotations, from the same participant, offer an interesting insight:

I compose primarily at the piano, although it is not my primary instrument. Rather, it serves as a tool to start the process of composing, and to confirm my musical intuitions when I’m feeling unsure. I spend hours trying out slightly different versions of the same short idea as I stitch things together. (P. 05)

Pencil and paper are an absolutely integral part of my process. The act of marking the page is one of the most important things I do as a composer. I have an almost obsessive need to “touch every note”, and to know that I’ve physically engaged with the material before it leaves the studio. (P. 05)

The first instance seems to point to an understanding of musical instruments as somewhat accessory. While the initial musical intuition can be verified on the piano, it has been arguably developed independently of it. The second part of the quotation, however, offers a different view: “stitching things together” at the piano is not just a verification of previously developed musical intuitions; instead, it is an integral part of the process. The same goes for tools such as pencil and paper, as confirmed by the second quotation: the interactions established between composers and their music are therefore not only figurative, but rather based on agency and bodily connections. As an example of how instrumental expertise informs composing more directly, consider the following:

As a pianist, I tend to write in “gestural counterpoint” also for monodic instruments such as the violin or the flute (for example action of the bow vs action of the left hand; or action of breath vs action on the keys). This often leads to a dissociation of the performers’ gestures in a way that reminds one of the relationship . . . between right and left hand on the piano. (P. 06)

Specific instrumental techniques can serve multiple functions, being adopted creatively to explore the expressive potential of different instrumental techniques. Interestingly, a composer’s expertise on the instrument(s) they have mastered can be generalized, leading to musical configurations with meaningful *traces* of it. This contributes to the creation of a palette of musical possibilities that often involve an intersubjective dimension:

I think a lot about how the music I compose will lie under the fingers of the performers. I have a good knowledge of how the standard orchestral instruments work, and I have a wonderful orchestration manual that I refer to often to ensure that what I compose will feel good for the player (though I also like to stretch their capacities as well). For example, I’ve composed many works for harp because I lived with a very inspiring harpist for four years in college. (P. 03)

Here our composer finds inspiration from the presence of others: the harpist who might have shared with them a number of useful tips about the instrument’s resources and techniques, and the performers who will probably play their piece. Indeed, even in cases where no instrumentalist is physically present, the quotation points to the desire of the composer – essential to the efficiency of their writing – to imagine just how the music is performed, and explore gestures and feelings that emerge as the performance unfolds. In the next section we will look more deeply at these how these relational occurrences are developed, and explore their role in shaping the composer’s activity.

### Establishing Relationships

This category captures the interplay of those social, physical, and historical aspects one can connect to, and enact, when composing music. We first examine these relationships from a general standpoint, consider how they are formed, and then explore how they affect one’s musical style and identity.

#### Synergies

Many composers are very protective of their work, as it often emerges from moments of focused isolation, personal struggle, and extended effort. Consider the following quotation:

I sometimes need space from others while I’m composing, I can be a bit of a loner when I’m in the groove. Other times I like to make music with others. Mostly I feel my compositional practice is just for me, and I don’t like to share it. (P. 03)

This echoes Mozart’s words, often cited: “when I am . . . completely myself, entirely alone, and of good cheer . . . it is on such occasions that my ideas flow best and most abundantly. Whence and how they come, I know not; nor can I force them” (quoted in Holmes, 2009, pp. 317). Yet, pragmatic considerations of musical composition process still raise the question: just how solitary can compositional activity really be? “I don’t buy the story of the composer being ‘isolated’ in an ivory tower. Being part of this historical period, external events – both personal and collective – highly affect my writing style” (P. 06). The same participant insisted that:

Composing means proposing to potential listeners – but also to performers – a sonic experience that could give rise to different reactions . . . This experience creates an ideal meeting point between the composer, from which the proposal starts, the performers, which mediates the proposal through interpretation, and the listeners, who are exposed to, and react to such proposal. (P. 06)

There is thus an ongoing dialogue between composers and their environment, one that mainly involves audience and performers. In a sense, “the audience is indirectly present during the composition process”^4^ (Zembylas & Niederauer, 2018, p. 22, quoted in Cook, 2018, p. 70), and so are the performers. Composers, in other words, are always exposed to, and shaped by, the rich webs of interactions they explore and build through their situated practice:

I’m inspired by many things; physical places (landscapes, buildings, environments both natural and constructed), abstract concepts such as “magnification”, “assembly”, “summation”, the visual arts, poetry, and dialogue with composers and musical traditions that preceded my own. (P. 05)

The synergies that composing brings into existence are thus varied and multifaceted. These include relationships between the composer and the physical location in which a piece is to be performed, imagined and actual performance, as well as music’s structural properties. These aspects are further highlighted in the following two quotations:

I always try to be connected to the extra-musical aspects of music performance. At the same time, I try to not let them overwhelm the musical aspects so that I try to let the music “work” even when the context changes. I also try to visualize the performance in the specific location so that, when is the case, I specify on the score the possible position of instruments on stage/location. (P. 04)

I get inspiration equally from “the notes themselves” (that is, just playing around on my piano, singing, or fiddling with electronic sounds until something interesting happens), or from abstract concepts (P. 05).

Both musical and extra-musical factors can thus play an important role in shaping creative trajectories and constructing broader musical ecologies. As another respondent put it: “My music is often programmatic^5^ (occasionally with multiple programmatic narratives under way simultaneously), so it is heavily related to people, things and situations” (P. 01). A final example of extra-musical factors includes various readings and ideas from others: “I have always fed on a variety of literature and philosophical and scientific writings. In general, however, I often find myself in ideas and authors which reflect my compositional act rather than getting inspiration from them” (P. 02). Note how this participant explicitly mentioned finding themselves in others’ ideas, rather than simply using them as inspirational resources. The interactivities emerging from the compositional act are situated in a shared ecology, which develops along with the composer’s artistic and personal flourishing. In what follows we investigate this two-way exchange at various levels, starting from the physical action of composing.

#### Feedback Loops

In what sense do bodily experience and music influence each other reciprocally in the act of composing? What kinds of extended relationships are brought into being as the creative process unfolds? In this code, we include experiences and descriptions that may help us put forward some preliminary answers. First, there is a basic sense in which bodily experience and music form a structured unity in the creative process, which involves the physical act of writing music. As we saw in the code *techniques and instruments*, this is an important factor in shaping one’s creative reach:

[Writing music with] pencil and paper influences the whole form [of a piece], because it allows me to draw diagrams [sketches] in a freedom, so that I can have an immediate grasp on gestures, dynamics, and general feeling. We could say that I have a visual approach to composition in its general form, because this gives me a global idea of the piece as I look at the sketch on paper. The physical act of writing music lets one reflect better on what one is doing, given that it forces one to go slower. (P. 06)

This quotation contextualizes the previous excerpt from another participant’s report, emphasizing the action of “marking the pages”. This is not only a ritual; instead, it is an integral part of the process that actively shapes the trajectories of the music being created. This is further emphasized in the case of computer-assisted writing. Consider the following reflection:

The computer is an instrument that really changed not only the way of writing music, but also to think about music . . . The computer generates a feedback process: for example, you could create a certain sound and then listen to it; if the timbre is not entirely convincing, you can think of another formalization to modify the previously obtained sound, and so on. (P. 02)

Gestures are important components of the creative process, and are often associated with various expressive, rhythmical, and timbral parameters, central to the piece being composed. The following two quotations illustrate how this can be so:

When I elaborate, and let instrumental compositional processes interact, I always presuppose executive gestures that correspond to the energy of the sound to be achieved. The articulations of rhythms are also gestural, like the piece’s orientations and tensions . . . Gestures are often unexpected, and translate into an actual choreography of generative sound actions. (P. 02)

I consider the gesture-sound association and reciprocal influence highly important. After all the performer’s movements on his or her instrument influence timbre and articulation. The physical work on the instrument, therefore, has an active role in creating a “sonic imaginary” which I then use for composing. (P. 06)

The feedback loops associated with gestures are also generalizable into principles and constraints that affect those mechanisms or rules one might give to their creative process. As one composer explained:

I associate movement with the concept of “floating section of a duration” as well as to the idea of energy; therefore, movement often suggests to me how to treat form; the energetic development [of the piece], and its articulation. But also, timbre, when movement is associated with a specific musical gesture. (P. 06)

Another participant reported being able to “think equally about instrumental gestures and about a kind of generalized dance movement” (P. 03). This can shape form at both the micro and macro level within the same piece: “I [often] conceive of my piece in its entirety as a series of general movements and, in parallel, I start deepening those movements . . . in a much more detailed way. This way, form at both micro and macro level influence each other recursively” (P. 06).

Having described how compositional practices often involve processes of discovery and co-transformation based on the development of relationships with things and people, we now explore how these also shape the composer’s musical identity.

#### Identities

In this code, we report considerations centred on musical identity and its development. One fundamental factor discussed here recalls again the relational aspects described in previous quotations: “[My musical identity] has evolved through a dialectic relationship with the composers I love, starting from those I have studied” (P. 07). While it seems that “the distribution of creativity across the living and the dead is the normal condition for classical music” (Cook, 2018, p. 65; see also Clarke & Doffman, 2017), particularly in historically informed performance, this quotation extends the argument to involve the notion of development. This is not understood in terms of a ladder, where personal growth follows a unidirectional trajectory. Instead, it is based on a continuous exchange, where individualities and collectivities are negotiated. This is also a main point in the following excerpt:

My musical identity is forged through listening. I am influenced by a broad range of styles and I try hard to stretch my ears. I love listening to music that is unfamiliar to me, and I love close listening. One of my most enduring, and confusing (to me), listening practices is getting hooked on a song and listening to it on repeat for days, until I wear it out, and hate it. I have increasingly incorporated this into my own works, paying close attention to the moments in my music that make me want to listen on repeat, and making them the core of the work. I’m fortunate to have had extremely permissive composition teachers, who let me experiment and be myself. (P. 03)

There is a tension between individual development and the incorporation of external influence, as if one could not really distinguish between the two in the creative process. And indeed, as another participant insisted, the role of the surrounding environment is essential:

I listened to thousands of hours of music. I studied at university and music college for the best part of a decade. I completed a PhD, in which I dissected and analyzed my work. I wrote many, many unsuccessful pieces . . . and then a few good ones. I chatted to my peers, my colleagues, my teachers, esteemed professional composers. I taught young composers. I let my outside interests inform my music. (P. 01)

This is backed up by another participant, who illustrated their idea of musical identity as follows:

Individual style mainly develops . . . through a mixture of instinct and method, relative to a cultural path made of experiences, thoughts, verbal exchanges, listening with other musicians and others . . . Thus, composing music, or as I prefer “to create”, entails a sense of invention that goes beyond my person. (P. 02)

This quotation points to the limits of individuality, and captures well the importance of external resources in shaping the composer’s developmental trajectory and style. In the next section we discuss the main findings of this study through the lenses of 4E cognitive science.

## General Discussion

Our qualitative study was designed to explore the complex range of experiences and feelings associated with the process of composing music in – broadly speaking – the Western, score-based tradition. The participants provided a number of insights which emphasize (i) the web of unfolding relationships associated with their practice, (ii) the importance of exploratory activity and musical discovery and (iii) the physicality involved in music-making and imagination. The focus on interpersonal relationships is particularly interesting when considering how Western classical music has been long seen as “the epitome of solo creativity” (Cook, 2018, p. 10), instantiated in individual geniuses. As we saw in the Introduction, this common view has been significantly challenged by novel research and theory that conceives of musical creativity as a social, collaborative phenomenon (e.g., Sawyer, 2006; van der Schyff et al., 2018), reflecting a more general shift in the cognitive sciences. In what follows, we highlight the importance of exploration, bodily experience and interaction by engaging with the different codes and categories described in the previous section.

The first aspect we investigated in the study was the definition of the key term *composing*. An important dimension that emerged was the emphasis on the power of music to help one either (re-)interpret, (re-)experience, and (re-)explore, different aspects of life; or create what Dibben and Haake (2013) name *auditory bubbles* – tools by which “one can escape or modify reality” (Tuuri & Peltola, 2019, p. 347). This process might be understood as a two-way exchange: not only does the act of musicking shape the sonic ecology in which we are embedded; it also provides a privileged access to an intimate sphere of personal experience. By creating new music our composers transform their environment as well as themselves. This resonates with insights offered by Brown and Dillon (2012). As we saw earlier, they maintain that creative acts allow composers to connect directly with their intuitive experience of music, leading to a meaningful engagement that plays out at both individual and social level. This requires the composer to be focused and absorbed in all aspects of the creative process, thereby generating meaning within the process rather than through its realization.

It then comes as no surprise that many participants associated the act of composing with a vast range of emotional states. Compositional practice, as described in the *self-reflections* code, is never neutral; it is a meaningful activity that brings together action, sounds, and feelings, reflecting the challenges and rewards associated with the creative process. This focus on the emotional dimension aligns with recent frameworks in creativity studies that emphasize the association of cognitive and emotional neural networks in the generation of creative ideas. For example, Dietrich (2004), put forward a comprehensive model of creativity based on functional neuroanatomy. Here the key role is played by the integration of four neural structures implementing the following functions: deliberate-cognitive; deliberate-emotional; spontaneous-cognitive; and spontaneous-emotional (see also Odena, 2018, p. 33). On this view, creative outcomes emerge from the interplay of these deliberate and spontaneous components and can occur in emotional or cognitive structures. Such a combination of deliberate and spontaneous processing is evident in our results, and challenges the view of artistic creativity as arising from sudden inspiration.

Moreover, from a 4E perspective, this focus on emotions and the *feeling body* (Colombetti, 2014) can be associated with action and interaction: since sensorimotor activity co-constitutes the mind, the sphere of affectivity – inseparable from its visceral and intersubjective roots – helps to shape mental life too. This view can open up interesting possibilities to rethink the generative role of emotions for musical outcomes. Consider how, in the *technique and instruments* code, the co-transformation of creative processes and products (a dichotomy that first appeared in the *definitions* code) emerged. As one proceeds in the act of composing, novel musical possibilities can be revealed, leading to unexpected outcomes and modification of existing schemas. Among the many variables that could shift the initial creative trajectory, the connection between composers and their instruments has inspired important contributions that look at how instruments can offer a variety of affordances that are both gestural and sonic, giving rise to a structured unity in perception and action (see De Souza, 2017; Windsor & de Bézenac, 2012). The relationship between composers and musical instruments was not described by all our participants in the same way, but takes on manifold forms. Some described their instruments as tools for inspiring or confirming novel musical ideas and intuitions; others linked their musical expertise to a more general gestural dimension. Yet, one way or another, a primacy of bodily experience seems to permeate the creative process. By way of example, we can reconsider Boden’s use of the term *conceptual space* (e.g., Boden, 2009). This involves a set of quasi-defined concepts to be explored by the creative agent (exploratory creativity), which are transformed when rules defining the artefacts that inhabit this space need to be changed (transformational creativity). While we agree with Boden that objects and items present in this conceptual space may be unknown in advance (for example, certain instrumental techniques or harmonic configurations may remain partially unknown before we have heard them), and may serve as tools to implement creative outcomes, we would argue that our data suggest that phases (explorations and transformation) are inseparable from the concrete activity in which musicking takes place, and drive relations between living systems. On this view, creative cognition involves the capacity to manipulate the environment physically, discover its regularities, explore its features, and establish relationships which transform its items as well as ourselves. This may bring to mind instances of the Geneplore model described above (Finke et al., 1992), particularly when considering the generation of novel ideas as being always context-dependent.

In the *synergies* code, we explored how “composers may construct meaning through a variety of engagements with music-making in personal and socio-cultural contexts” (Brown & Dillon, 2012, p.105). Most of our participants describe the process of composing music as an immersion in a community of practice. This once again highlights the role of interaction for shaping creative thought and action, showing strong commonalities with work in enactive cognitive science focused on *participatory sense-making*. This is “the coordination of intentional activity in interaction, whereby individual *sense*-*making* processes are affected, and new domains of social *sense*-*making* can be generated that were not available to each individual on her own” (De Jaegher & Di Paolo, 2007, p. 497, authors’ italics). However, not only do social others take part in meaning generation; physical features of the environment such as computers can also contribute to the shaping the composer’s musical intuitions. This interactivity illustrates very well how “the materials with which composers work ‘talk back’ to them” (Cook, 2018, p. 11), a view that differs in important respects from more traditional accounts such as that put forward by Wallas (1926), in which external information is thought to be acquired at the very beginning of the process and then elaborated internally.

Instead, the interplay between internal and external factors forges a series of *feedback loops*, whereby existing relationships are re-developed and re-discovered in the act of composing: the physical act of rendering a piece of music as a musical score is an action open to possible changes and fluctuations in the intentions that guide it. The creative product is not just an outcome of internal processes of decision-making; instead it reflects the relational nature of its underlying processes: writing with a computer or with pencil and paper arguably leads to different bodily states and musical explorations, as both tools have physical properties that are distinctively coupled with the composer in various ways. This suggests that, in composing a piece of music, part of the creative process is offloaded to the tools used by the composer for writing, and relies on the bodily experience it entails. Such a view aligns with work on the extended mind described above, where living systems are seen to use biological and non-biological information and tools in a functionally similar way. In music, this approach has been applied to the context of music-making and listening (e.g., Cochrane, 2008; Ryan & Schiavio, 2019), but, as far as we know, has not been applied to composition.

The focus on *identities* offers a good opportunity to reflect on the different timescales involved in compositional activity. Experienced composers have usually many years of training, often based on style exercises (e.g., composing polyphonic vocal music in the style of Palestrina; a four-part fugue in the style of Bach, a *lied* in the style of Schubert, etc.), theoretical knowledge (e.g., music theory, psychoacoustics, harmony, etc.), and practical insights (e.g., orchestration, instrumentation, etc.). This might explain why one of our participants referred to a constant dialogue with “past composers”. However, others have noticed how interactions with styles and genres they like, as well as engagement in discussions with performers and other composers, can play an important role in shaping their identity. This apparent dichotomy recalls the model put forward by Folkestad (2012):

There two levels of collective communication in the process of collective, creative music-making: (1) one interpersonal, or “interpsychological” functioning [. . .], between the individuals of the working group of the collective activity, and also (2) one intrapersonal, or “intrapsychological” [. . .], a dialogue with the collective experiences and knowledge of previous composers mediated by the tools in use. The latter also constitutes the collective dimension in “individual” activities. (Folkestad, 2012, p. 198)

The web of interactivities developed by the composer provides a platform for the creative process to unfold. In a sense, the development of relevant relationships becomes a condition of possibility for creative work rather than a property or quality of the creative output. And, as pointed out by Folkestad, there is a *collective* dimension to *individual* practice that plays a specific role in both compositional activity and in the development of musical identity (see Høffding & Satne, 2019). This is sedimented in our history of structural couplings with the environment; develops through novel assemblies and relations; and can be re-discovered and interpreted through action and exploration. As one participant put it, composing music “entails a sense of invention”, which “goes beyond” the individual. Musical identities seem here to reflect such a dialogical description, providing further grounding for the thesis that “creativity and musical creativity are developmental concepts and, given the appropriate holding environment, they can be nurtured and developed by everybody” (Odena, 2018, p. 144). From the perspective that focuses on the products of creative activity, such insights echo previously mentioned accounts in which creative outputs illustrate an optimal balance between originality and appropriateness (Runco & Jaeger, 2012). The *dialogues* that take place between composers and their past and future encounters may contribute to both the innovativeness of a new piece of music while its style and form are nevertheless recognizable.

Before concluding, we note a main limitation to our study. The high degree of our participants’ expertise as performers may account for their focus on action and physical engagement as reported in their responses to the questionnaire; all participants were trained instrumentalists, and this may have biased the results. Another aspect worth mentioning is that, while most of the results align closely with the core concepts of the 4E approach, some may resonate with a more traditional view. For instance, when P. 06 maintained that performers mediate an initial proposal of the composer through interpretation, with listeners passively reacting to it, they portrayed a rather linear schema of musical communication – one that gives members of the audience no causal role in the process of composition. However, in another quotation they mentioned that their writing is strongly inspired by a range of social and cultural factors (as they put it, composers do not operate in an “ivory tower”). Such external aspects may include members of the audience too, but ultimately it remains unclear in what sense they actually take part in the process. Future work may thus specifically explore the extent to which composers draw on anticipated interactions with others to inform their musical ideas and what differences emerge when comparing memories of past interactions with imaginative reflections on future connections with concertgoers, performers, and other composers.

## Conclusion

Our study suggests that creative cognition in score-based composition involves three overlapping characteristics: it is exploratory, it is grounded in bodily experience, and it emerges from the recursive dialogue of agents with their social, cultural, and physical environments. As such, forms of musical creativity involved in the deliberate creation of novel musical material might be understood as processes whereby composers enact their musical identities by exploring novel interactivities within their contingent and historical milieux. Recent work by Tuuri and Peltola (2019) offers a similar account of musical imagination. Inspired by work by Gallagher (2000; 2017), they argue that imagining music involves the enactment of sedimented experiences based on the recursive interplay of self-agency and the narrative dimension of creative thought. As both aspects build on our histories of social and musical interactions with the environment, the “generative processes of imaging are not only individual but also exhibited and jointly engaged in social dialogues” (Tuuri & Peltola, 2019, p. 354). In this article we presented conceptual and qualitative-based arguments that extend these insights, aiming to develop a similar understanding of composition by capturing the complex interplay of bodily, exploratory, and interactive elements in composers and their practices. While our study is necessarily limited, being framed within the constraints of Western compositional practice, it nevertheless offers an opportunity to reflect upon the cognitive foundations of musical creativity, and invites further discussion and exchanges with scholars, performers, and composers. In doing so it aligns with recent research on creativity, cognitive science, and musical imagination, which takes seriously the challenge of developing a more embodied, exploratory, and relational story of what mental life and musicking entail.

## Supplemental Material

Resub_Appendix_v1.1 – Supplemental material for Processes and Experiences of Creative Cognition in Seven Western Classical Composers Click here for additional data file. Supplemental material, Resub_Appendix_v1.1 for Processes and Experiences of Creative Cognition in Seven Western Classical Composers by Andrea Schiavio, Nikki Moran, Dylan van der Schyff, Michele Biasutti and Richard Parncutt in Musicae Scientiae

## Notes

1. Tools and musical instrument may become part of such an extended system for some cognizers but not for others. Expert performers, for example, know how to optimize their relationship with the instrument in ways that novices are arguably unaware of. Consider how a novice guitar player may spend considerable time to make sure each finger of the left hand is in the right position when playing a chord, whereas experts display the ability to play a chord without focusing on each finger. During an improvised solo, this ability allows experts to explore a vaster range of possibilities of the instrument (e.g., related to articulation, timbre, expressivity, etc.), as if they were “thinking” through the instrument (see also Malafouris, 2020).

2. According to Nagy (2017), this entails a “creative space that in part resembles musical improvisation, whose psychological contingencies give rise to a sense of embodied experience” (Nagy, 2017, p. 88).

3. Here, and in other cases, our participants appear to report a sense of absorption taking part in the composition process. While a number of studies in music, philosophy, and in the arts focus on this phenomenon in performance (e.g., Bergamin, 2017; Høffding, 2019; Montero, 2016), or perception (e.g., Sandstrom & Russo, 2013; Schubert et al., 2018), less is known about absorption during music composition (but see Chirico et al., 2015, and MacDonald et al., 2006). Where does it come from? And when is it more likely to arise? We do not have answers to these questions emerging from our data, so we can only speculate that the task of composing music can lead to absorption-like states when a certain connection with music is established – one that transforms the composer as well as their musical outcomes. It is perhaps in this reciprocal interplay that the composer may feel lost in the process, sharing with music itself the control over different musical possibilities, choices and actions.

4. As Cook insists, “Mozart was composing people responses as much as he was composing the notes” (Cook, 2018, p. 70).

5. We re-contacted P. 01 via email and asked them to clarify this statement. They replied: “In this context, what I meant by ‘programmatic’ music is music that uses an extra-musical narrative stimulus (a story, a poem, etc.), and that allows this extra-musical narrative stimulus to impact upon the music.”

## References

[bibr1-1029864920943931] Abraham A. (2018). The neuroscience of creativity. Cambridge University Press.

[bibr2-1029864920943931] Bailey B. (1993). Improvisation: Its nature and practice in music. Da Capo.

[bibr3-1029864920943931] Barrett M. (1998). Researching children’s compositional process and products: connection to music education practice. In Sundrin B. MacPherson G.E. Folkestad G. (Eds.), Children composing (pp. 10–35). Malmo Academy of Music.

[bibr4-1029864920943931] Bennett J. (2012). Constraints, collaboration, and creativity, in popular songwriting teams. In Collins D. (Ed.), The Act of musical composition – Studies in the creative process (pp. 139–169). Ashgate.

[bibr5-1029864920943931] Bergamin J. A. (2017). Being-in-the-flow: Expert coping as beyond both thought and automaticity. Phenomenology and the Cognitive Sciences, 16(3), 403–424.

[bibr6-1029864920943931] Biasutti M. (2013). Orchestra rehearsal strategies: conductor and performer views. Musicae Scientiae, 17(1), 57–71. 10.1177/1029864912467634

[bibr7-1029864920943931] Biasutti M. (2015). Creativity in virtual spaces: Communication modes employed during collaborative online music composition, Thinking Skills and Creativity, 17, 117–129.

[bibr8-1029864920943931] Biasutti M. (2018). Strategies adopted during collaborative online music composition, International Journal of Music Education, 36(3), 473–490. 10.1177/0255761417741520

[bibr9-1029864920943931] Bishop L. (2018). Collaborative musical creativity: How ensembles coordinate spontaneity. Frontiers in Psychology, 9:1285. 10.3389/fpsyg.2018.01285 30087645 PMC6066987

[bibr10-1029864920943931] Boden M. (2004). The creative mind. Myths and mechanisms (2nd edition). Routledge.

[bibr11-1029864920943931] Boden M. (2009). Conceptual spaces. In Meusburger P. Funke J. Wunder E. (Eds.), Milieus of creativity. Knowledge and space, Vol. 2 (pp. 235–243). Springer.

[bibr12-1029864920943931] Bowman W. (2004). Cognition and the body: Perspectives from music education. In Bresler L. (Ed.), Knowing bodies, moving minds: Towards embodied teaching and learning (pp. 29–50). Kluwer Academic Press.

[bibr13-1029864920943931] Brown A. R. Dillon S. (2012). Meaningful engagement: Creative experiences with music composition. In Collins D. (Ed.), The Act of musical composition – Studies in the creative process (pp. 79–110). Ashgate.

[bibr14-1029864920943931] Burnard P. (1999). Bodily intention in children’s improvisation and composition. Psychology of Music, 27(2). 159–174. 10.1177/0305735699272007

[bibr15-1029864920943931] Burrows J. (2004). Musical archetypes and collective consciousness: Cognitive distribution and free improvisation. Critical Studies in Improvisation, 1(1). 10.21083/csieci.v1i1.11

[bibr16-1029864920943931] Canonne C. Garnier N. B. (2015). Individual decisions and perceived form in collective free improvisation. Journal of New Musical Research, 44(2), 145–167.

[bibr17-1029864920943931] Chemero A. (2009). Radical embodied cognitive science. MIT Press.

[bibr18-1029864920943931] Chirico A. Serino S. Cipresso P. Gaggioli A. Riva G. (2015). When music “flows”. State and trait in musical performance, composition and listening: a systematic review. Frontiers in Psychology: 6:906. 10.3389/fpsyg.2015.00906 26175709 PMC4485232

[bibr19-1029864920943931] Clark A. (2008). Supersizing the mind: Embodiment, action and cognitive extension. Oxford University Press.

[bibr20-1029864920943931] Clark A. Chalmers D. (1998). The extended mind. Analysis, 58(1), 7–19.

[bibr21-1029864920943931] Clarke E. Doffman M. (Eds.) (2017), Distributed creativity: Collaboration and improvisation in contemporary music. Oxford University Press.

[bibr22-1029864920943931] Cochrane T. (2008). Expression and extended cognition. The Journal of Aesthetics and Art Criticism, 66(4), 59–73.

[bibr23-1029864920943931] Collins D. (2005). A synthesis process model of creative thinking in music composition. Psychology of Music, 33(2), 193–216. 10.1177/0305735605050651

[bibr24-1029864920943931] Colombetti G. (2014). The feeling body: Affective science meets the enactive mind. MIT Press.

[bibr25-1029864920943931] Cook N. (2018). Music as creative practice. Oxford University Press.

[bibr26-1029864920943931] Cross I. (2003). Music and biocultural evolution. In Clayton M. Herbert T. Middleton R. (Eds), The cultural study of music: A critical introduction (pp. 19–30). Routledge.

[bibr27-1029864920943931] De Jaegher H. Di Paolo E. (2007). Participatory sense-making: An enactive approach to social cognition. Phenomenology and the Cognitive Sciences, 6(4), 485–507.

[bibr28-1029864920943931] De Souza J. (2017). Music at hand: Instruments, bodies, and cognition. Oxford University Press.

[bibr29-1029864920943931] Dean R. T. (2017), Creating music: Composition. In Ashley R. Timmers R. (Eds.), The Routledge companion to music cognition (pp. 251–263). Routledge.

[bibr30-1029864920943931] Demos A. P. Chaffin R. Logan T. (2018). Musicians body sway embodies musical structure and expression: A recurrence-based approach. Musicae Scientiae, 22(2), 244–263.

[bibr31-1029864920943931] Di Paolo E. Buhrmann T. Barandiaran X. E. (2017). Sensorimotor life: An enactive proposal. Oxford University Press.

[bibr32-1029864920943931] Dibben N. Haake A.B. (2013). Music and the construction of space in office-based work settings. In Born G. (Ed.). Music, sound and space: Transformations of public and private experience (pp. 151–168). Cambridge University Press.

[bibr33-1029864920943931] Dietrich A. (2004). The cognitive neuroscience of creativity. Psychonomic Bulletin & Review, 11(6), 1011–1026. 10.3758/BF03196731 15875970

[bibr34-1029864920943931] Fachner J. (2008). Musik und veränderte Bewusstseinszustände. In Bruhn H. Kopiez R. Lehmann A.C. (Eds.) Musikpsychologie. Das neue Handbuch (pp. 594–612). Rowohlt.

[bibr35-1029864920943931] Finke R. A. Ward T. B. Smith S. M. (1992). Creative cognition. MIT Press.

[bibr36-1029864920943931] Folkestad G. (2012). Digital tools and discourse in music: The ecology of composition. In Hargreaves D. J. Miell D. E. MacDonald R. A. R. (Eds.) Musical imaginations. (pp.193–205). Oxford University Press.

[bibr37-1029864920943931] Frith S. (1996). Performing rites. On the value of popular music. Oxford University Press

[bibr38-1029864920943931] Fuchs T. (2018). Ecology of the brain: The phenomenology and biology of the embodied mind. Oxford University Press.

[bibr39-1029864920943931] Gallagher S. (2000). Philosophical conceptions of the self: Implications for cognitive science. Trends in Cognitive Sciences, 4(1), 14–21. 10637618 10.1016/s1364-6613(99)01417-5

[bibr40-1029864920943931] Gallagher S. (2017). Enactivist interventions: Rethinking the mind. Oxford University Press.

[bibr41-1029864920943931] Hill J. (2018). Becoming creative: Insights from musicians in a diverse world. Oxford University Press.

[bibr42-1029864920943931] Høffding S. Schiavio A. (2019). Exploratory expertise and the dual intentionality of musicmaking. Phenomenology and the Cognitive Sciences. 10.1007/s11097-019-09626-5 PMC8570311 34759788

[bibr43-1029864920943931] Høffding S. (2019). A phenomenology of musical absorption. Palgrave Macmillan.

[bibr44-1029864920943931] Høffding S. Satne G. (2019). Interactive expertise in solo and joint musical performance. Synthese. Advance online publication. 10.1007/s11229-019-02339-x

[bibr45-1029864920943931] Holmes E. (2009). The life of Mozart, including his correspondence. Cambridge University Press.

[bibr46-1029864920943931] Hosseini S. Deng X. Miyake Y. Nozawa T. (2019). Head movement synchrony and idea generation interference. Investigating background music effects on group creativity. Frontiers in Psychology, 10:2577. 10.3389/fpsyg.2019.02577 31803115 PMC6873777

[bibr47-1029864920943931] Johansen G. G. Nielsen S. G. (2019) The practicing workshop: A development project. Frontiers in Psychology, 10:2695. 10.3389/fpsyg.2019.02695. 31866901 PMC6910069

[bibr48-1029864920943931] Kenett Y. N. Anaki D. Faust M. (2014). Investigating the structure of semantic networks in low and high creative persons. Frontiers in Human Neuroscience, 8, 407. 10.3389/fnhum.2014.00407 24959129 PMC4051268

[bibr49-1029864920943931] Kenny A. (2016). Communities of musical practice. Routledge.

[bibr50-1029864920943931] Kratus J. K. (1989). A time analysis of the compositional processes used by children aged 7 to 11. Journal of Research in Music Education, 37(1), 5-20.

[bibr51-1029864920943931] Krueger J. (2013). Empathy, enaction, and shared musical experience. In Cochrane T. Fantini B. Scherer K. (Eds.), The emotional power of music: Multidisciplinary perspectives on musical expression, arousal, and social control (pp. 177–196). Oxford University Press.

[bibr52-1029864920943931] Lakoff G. Johnson M . (1980). Metaphors We Live by. Chicago University Press.

[bibr53-1029864920943931] Leman M. (2007). Embodied music cognition and mediation technology. MIT Press.

[bibr54-1029864920943931] Leman M. Maes P.-J. (2016). The role of embodiment in the perception of music. Empirical Musicology Review, 9(3–4), 236–246. 10.18061/emr.v9i3-4.4498

[bibr55-1029864920943931] Linson A. Clarke E. (2017). Distributed cognition, ecological theory and group improvisation. In Clarke E. M. Doffman M. (Eds.) Distributed creativity: Collaboration and improvisation in contemporary music (pp. 233–241). Oxford University Press.

[bibr56-1029864920943931] Loaiza J. M. (2016). Musicking, embodiment and the participatory enaction of music: outline and key points. Connection Science, 28, 410–422. 10.1080/09540091.2016.1236366

[bibr57-1029864920943931] MacDonald R. Miell D. Morgan L. (2000). Social processes and creative collaboration in children. European Journal of Psychology of Education, 15, 405–415. 10.1007/BF03172984

[bibr58-1029864920943931] MacDonald R. Byrne C. Carlton L. (2006). Creativity and flow in musical composition: an empirical investigation. Psychology of Music, 34(3), 292–306. 10.1177/0305735606064838

[bibr59-1029864920943931] Mackinnon D. W. (1965). Personality and the realization of creative potential. American Psychologist, 20(4), 273–281. 10.1037/h0022403 14268806

[bibr60-1029864920943931] Malafouris L. (2013). How things shape the mind: A theory of material engagement. MIT Press.

[bibr61-1029864920943931] Malafouris L. (2015). Metaplasticity and the primacy of material engagement. Time and Mind, 8(4), 351–371. 10.1080/1751696X.2015.1111564

[bibr62-1029864920943931] Malafouris L. (2020). Thinking as “thinging”: Psychology with things. Current Directions in Psychological Science, 29(1), 3–8. 10.1177/0963721419873349

[bibr63-1029864920943931] Malloch S. Trevarthen C. (2018). The human nature of music. Frontiers in Psychology, 9:1680. 10.3389/fpsyg.2018.01680 30337892 PMC6180173

[bibr64-1029864920943931] Mednick S. (1962). The associative basis of the creative process. Psychological Review, 69(3), 220–232. 14472013 10.1037/h0048850

[bibr65-1029864920943931] Mithen S. (2006). The singing Neanderthals: The origins of music, language, mind, and body. Harvard University Press.

[bibr66-1029864920943931] Montero B. G. (2016). Thought in action: Expertise and the conscious mind. Oxford University Press.

[bibr67-1029864920943931] Montuori A. Purser R. E. (1995). Deconstructing the lone genius myth: Toward a contextual view of creativity. Journal of Humanistic Psychology, 35(3), 69–112.

[bibr68-1029864920943931] Moran N. (2014). Social implications arise in embodied music cognition research which can counter musicological “individualism”. Frontiers in Psychology, 5:676. 10.3389/fpsyg.2014.00676 25101011 PMC4102907

[bibr69-1029864920943931] Morgan L. (1999). Children’s collaborative music composition: Communication through music. Unpublished doctoral dissertation, University of Leicester, UK.

[bibr70-1029864920943931] Morrison S. J. Demorest S. M. Aylward E. H. Cramer S. C. Maravilla K. R. (2003). fMRI investigation of cross-cultural music comprehension. NeuroImage, 20(1), 378–384. 14527597 10.1016/s1053-8119(03)00300-8

[bibr71-1029864920943931] Morrison S. J. Demorest S. M. Stambaugh L.A. (2008). Enculturation effects in music cognition: The role of age and music complexity. Journal of Research in Music Education, 56(2), 118–129.

[bibr72-1029864920943931] Nagy Z. (2017). Embodiment of musical creativity: The cognitive and performative causality of musical composition. Routledge.

[bibr73-1029864920943931] Newen A. De Bruin L. Gallagher S. (2018). The Oxford handbook of 4E cognition. Oxford University Press.

[bibr74-1029864920943931] Nijs L. (2017). The merging of musician and musical instrument: Incorporation, presence and the levels of embodiment. In Lesaffre M. Maes P. J. Leman M. (Eds.). The Routledge companion to embodied music interaction (pp. 49–57). Routledge.

[bibr75-1029864920943931] Odena O. (2018) Musical creativity revisited: Educational foundations, practices and research. Routledge.

[bibr76-1029864920943931] Overy K. Molnar-Szakacs I. (2009). Being together in time: Musical experience and the mirror neuron system. Music Perception, 26(5), 489–504. 10.1525/mp.2009.26.5.489

[bibr77-1029864920943931] Oyama S. (2000). The ontogeny of information: Developmental systems and evolution. Duke University Press.

[bibr78-1029864920943931] Reybrouck M. (2005). A biosemiotic and ecological approach to music cognition: event perception between auditory listening and cognitive economy. Axiomates, 15, 391–409. 10.1007/s10516-004-6679-4.

[bibr79-1029864920943931] Reybrouck M. (2012). Musical sense-making and the concept of affordance: an ecosemiotic and experiential approach. Biosemiotics, 5, 391–409. 10.1007/s12304-012-9144-6

[bibr80-1029864920943931] Runco M. Jaeger G. (2012) The standard definition of creativity, Creativity Research Journal, 24(1), 92–96. 10.1080/10400419.2012.650092

[bibr81-1029864920943931] Ryan K. Schiavio A. (2019). Extended musicking, extended mind, extended agency. Notes on the third wave. New Ideas in Psychology, 55, 8–17. 10.1016/j.newideapsych.2019.03.001

[bibr82-1029864920943931] Sandstrom G. M. Russo F. A. (2013). Absorption in music: Development of a scale to identify individuals with strong emotional responses to music. Psychology of Music, 41(2), 216–228. 10.1177/0305735611422508

[bibr83-1029864920943931] Sawyer R. K. (2006). Group creativity: Musical performance and collaboration. Psychology of Music, 34(2), 148–165. 10.1016/j.newideapsych.2019.03.001

[bibr84-1029864920943931] Sawyer R. K. DeZutter S. (2009). Distributed creativity: How collective creations emerge from collaboration. Psychology of Aesthetics, Creativity, and the Arts, 3(2), 81–92.

[bibr85-1029864920943931] Seddon F. Biasutti M. (2009). A comparison of modes of communication between members of a string quartet and a jazz sextet. Psychology of Music, 37(4), 395–415. 10.1177/0305735608100375

[bibr86-1029864920943931] Schäfer T. Sedlmeier P. Städtler C. Huron D. (2013). The psychological functions of music listening. Frontiers in Psychology, 4, 511. 10.3389/fpsyg.2013.00511 23964257 PMC3741536

[bibr87-1029864920943931] Schiavio A. Altenmüller E. (2015). Exploring music-based rehabilitation for Parkinsonism through embodied cognitive science. Frontiers in Neurology, 6:217. 10.3389/fneur.2015.00217 26539155 PMC4609849

[bibr88-1029864920943931] Schiavio A. Timmers R. (2016). Motor and audiovisual learning consolidate auditory memory of tonally ambiguous melodies. Music Perception, 34(1), 21–32.

[bibr89-1029864920943931] Schiavio A. Biasutti M. van der Schyff D. Parncutt R. (2018). A matter of presence. A qualitative study on teaching individual and collective music classes. Musicae Scientiae. OnlineFirst. 10.1177/1029864918808833

[bibr90-1029864920943931] Schiavio A. Gesbert V. Reybrouck M. Hauw D. Parncutt R. (2019). Optimizing performative skills in social interaction. Insights from embodied cognition, music education, and sport psychology. Frontiers in Psychology, 10:1542. 10.3389/fpsyg.2019.01542 31379644 PMC6646732

[bibr91-1029864920943931] Schiavio A. Stupacher J. Parncutt R. Timmers R. (2020). Learning music from each other. Synchronization, turn-taking or imitation? Music Perception, 37(5), 403–422.

[bibr92-1029864920943931] Schiavio A. van der Schyff D. Gande A. Kruse-Weber S. (2019). Negotiating individuality and collectivity in community music. A qualitative case study. Psychology of Music, 47(5), 706–721. 10.1177/0305735618775806

[bibr93-1029864920943931] Schubert E. Halpern A. R. Kreutz G. Garrido S. (2018). Attraction to sad music: The role of imagery, absorption, and rumination. Psychology of Aesthetics, Creativity, and the Arts, 12(3), 251–258. 10.1037/aca0000160

[bibr94-1029864920943931] Shapiro L . (2011). New problems of philosophy. Embodied cognition. Routledge/Taylor & Francis Group.

[bibr95-1029864920943931] Sloboda J. (1985). The musical mind: The cognitive psychology of music. Clarendon.

[bibr96-1029864920943931] Sloboda J. (1988). Generative processes in music: The psychology of composition, performance, and improvisation. Clarendon.

[bibr97-1029864920943931] Small C. (1998). Musicking: The meaning of performing and listening. Wesleyan University Press.

[bibr98-1029864920943931] Thompson E. (2007). Mind in life: Biology, phenomenology, and the sciences of mind. Harvard University Press.

[bibr99-1029864920943931] Toop D. (2016). Into the maelstrom: Music, improvisation and the dream of freedom. Bloomsbury.

[bibr100-1029864920943931] Turino T. (2008). Music as social life: The politics of participation. University of Chicago Press.

[bibr101-1029864920943931] Tuuri K. Peltola H.-R. (2019). Building worlds together with sound and music: Imagination as an active engagement between ourselves. In Grimshaw-Aagaard M. Walther-Hansen M. Knakkergaard M. (Eds.), The Oxford handbook of sound and imagination, Vol. 1 (pp. 345–358). Oxford University Press.

[bibr102-1029864920943931] van der Schyff D. Schiavio A. Walton A. Velardo V. Chemero A. (2018). Musical creativity and the embodied mind: Exploring the possibilities of 4E cognition and dynamical systems theory. Music & Science. 10.1177/2059204318792319.

[bibr103-1029864920943931] Varela F. Thompson E. Rosch E. (1991). The embodied mind: Cognitive science and human experience. MIT Press.

[bibr104-1029864920943931] Wallas G. (1926). Art of thought. Harcourt-Brace.

[bibr105-1029864920943931] Walton A. Richardson M. J. Chemero A. (2014). Self-organization and semiosis in jazz improvisation. International Journal of Signs and Semiotic Systems, 3(2), 12–25.

[bibr106-1029864920943931] Walton A. Washburn A. Langland-Hassan P. Chemero A. Kloos H. Richardson M. J. (2018). Creating time: Social collaboration in music improvisation. Topics in Cognitive Science, 10(1), 95–119. 10.1111/tops.12306 29152904 PMC5939966

[bibr107-1029864920943931] Webster P.R. (1992) Research on creative thinking in music: The assessment issue. In Colwell R. (Ed.) Handbook of research on music teaching and learning (pp. 226–280). Schirmer.

[bibr108-1029864920943931] Wenger E. (2002). Cultivating communities of practice: a guide to managing knowledge. Harvard University Press.

[bibr109-1029864920943931] Windsor W. L. de Bézenac C. (2012). Music and affordances. Musicae Scientiae, 16(1), 102–120. 10.1177/1029864911435734

[bibr110-1029864920943931] Zembylas T. Niederauer M. (2018) Composing processes and artistic agency: Tacit knowledge in composing. Routledge.

