# Supplementary material for: Processes and Experiences of Creative Cognition in Seven Western Classical Composers
Source: Music Sci. 2020 Aug 8;26(2):303–25. doi: 10.1177/1029864920943931 (PMC9082970; doi:10.1177/1029864920943931)
Supplement: Resub_Appendix_v1.1 – Supplemental material for Processes and Experiences of Creative Cognition in Seven Western Classical Composers [file Resub_Appendix_v1.1.pdf]

Please provide detailed answers to the questions below, without leaving out anything - including aspects that might be considered trivial. This questionnaire is completely anonymous. It does not evaluate aspects related to intelligence or musical competence. It only aims to gather information on how you think about and experience your work as a composer.

1. What does ‘composing music’ mean to you?

2. What inspires you when composing?

3. On what principles do you 'translate' inspiration into music?

4. How do you feel when composing music?

5. Have you ever thought of associating your music with visual aspects such as lights or images?

6. What is the role of improvisation in your compositions?

7. What role does instrumental practice play when composing

8. How do you relate to people, things, and situations in your compositional practice?

9. When you compose music, do you think about the eventual client (or commissioning organization), the location where it will be performed, the context, and the audience? How do these influence your writing?

10. What tools and devices do you use to compose music?

11. How do computers, pen and paper, and instruments shape your writing respectively?

12. What role do the performers' gestures play in influencing your writing?

13. When you compose music, do you think more about movement in general or more about specific (e.g., instrumental) gestures?

14. How much does the familiarity with gestures specific to your instrument inform your compositional style?

15. How did you develop your own personal musical identity and style?
